# Insomnia and Relationship with Anxiety in University Students: A Cross-Sectional Designed Study

**DOI:** 10.1371/journal.pone.0149643

**Published:** 2016-02-22

**Authors:** Nour Choueiry, Tracy Salamoun, Hicham Jabbour, Nada El Osta, Aline Hajj, Lydia Rabbaa Khabbaz

**Affiliations:** 1 Faculty of Pharmacy, Saint-Joseph University of Beirut, Beirut, Lebanon; 2 Department of Anesthesia and Critical Care, Hôtel-Dieu de France Hospital, Saint-Joseph University of Beirut, Beirut, Lebanon; 3 Faculty of Medicine, Saint-Joseph University of Beirut, Beirut, Lebanon; 4 Department of Public Health, Faculty of Medicine, Saint-Joseph University, Beirut, Lebanon; 5 Department of Prosthodontics, Faculty of Dental Medicine, Saint-Joseph University, Beirut, Lebanon; 6 Laboratoire de Pharmacologie, Pharmacie clinique et Contrôle de qualité des médicaments, Faculty of Pharmacy, Saint-Joseph University of Beirut, Beirut, Lebanon; University of Rome Tor Vergata, ITALY

## Abstract

**Purpose:**

Sleep disorders (SDs) are now recognized as a public health concern with considerable psychiatric and societal consequences specifically on the academic life of students. The aims of this study were to assess SDs in a group of university students in Lebanon and to examine the relationship between SDs and anxiety.

**Methods:**

An observational cross-sectional study was conducted at Saint-Joseph University, Lebanon, during the academic year 2013–2014. Four questionnaires were face-to-face administered to 462 students after obtaining their written consent: Insomnia Severity Index (ISI), Pittsburgh Sleep Quality Index (PSQI), Epworth Sleepiness Scale (ESS), and Generalized Anxiety Disorder 7-item scale (GAD-7).

**Results:**

The prevalence of clinically significant insomnia was 10.6% (95% CI: 7.8–13.4%), more frequent in first year students. ISI mean score was 10.06 (SD = 3.76). 37.1% of the participants were poor sleepers. Excessive daytime sleepiness (EDS) and poor sleep were significantly more frequent among participants with clinical insomnia (*p* = 0.031 and 0.001 respectively). Clinically significant anxiety was more frequent in students suffering from clinical insomnia (*p* = 0.006) and in poor sleepers (*p* = 0.003). 50.8% of the participants with clinically significant anxiety presented EDS versus 30.9% of those with no clinically significant anxiety (*p*<0.0001).

**Conclusions:**

The magnitude of SDs in this sample of Lebanese university students demonstrate the importance of examining sleep health in this population. Moreover, the link between SD and anxiety reminds us of the importance of treating anxiety as soon as detected and not simply targeting the reduction of sleep problems.

## Introduction

The American National Commission of Sleep Disorders Research (NCSDR) defines insomnia as “a chronic or acute sleep disorder characterized by a complaint of difficulty initiating, and/or maintaining sleep, and/or a subjective complaint of poor sleep quality that result in daytime impairment and subjective report of impairment” [1].

The different sets of questions and criteria used to assess and define insomnia result in a wide range of prevalence rates [2]. A general consensus has emerged from population-based studies. This consensus states that approximately 30% of a variety of adult samples from different countries have at least one of the symptoms of insomnia, such as difficulty initiating or maintaining sleep, waking up too early, and in some cases, having non-restorative or poor quality of sleep [3]. The DSM-IV-TR and research diagnostic criteria/International Classification of Sleep disorders, 2^nd^ Edition (RDC/ICSD-2) are commonly used diagnostic systems. These research tools have difficulties initiating or maintaining sleep in addition to daytime distress or impairment, but they differ in other symptoms required for diagnosis [2]. Recent studies have suggested that sleep dissatisfaction may be an important indicator of sleep pathology [4] and in the newly published DSM-5, dissatisfaction with sleep quality and quantity has been included as a necessary condition for insomnia diagnosis; in addition, the frequency of sleep problems for at least 3 nights per week and during a period of at least three months are required [5]. Insomnia is now recognized as a public health concern that affects the quality of life of millions of people around the world as it can lead to long-term physical and mental exhaustion with altered mood, concentration, and memory. Subsequently, the social and professional aspects of life are affected because of a deterioration of general condition with a decrease in intellectual abilities and cognitive behavior [6]. Sleep disorders (SDs) are particularly increasing in students as they face multiple stressors such as academic overload, constant pressure to succeed, and concerns about the future that alter the quality of their sleep. Socio-demographic factors such as age and gender, sleeping hygiene, physical illnesses and mental disorders were identified as the main correlates of insomnia [7]. Individuals with family histories of depression or anxiety and who manifest lifelong depression and anxiety beginning in childhood are at uniquely high risk for insomnia at midlife [8]. Moreover, a considerable number of researches documented a high prevalence of sleep problems among clinically-anxious youth [9, 10] as well as elevated levels of anxiety and stress in university students [11]. A few studies suggest that anxiety may be a risk factor for future insomnia [12, 13] and many suggest a bidirectional relationship between anxiety (and depression) and insomnia [14, 15]. Among anxiety disorders, GAD (generalized anxiety disorder) has been identified as the disorder with the highest comorbidity of sleeping problems/insomnia [16].

Given the seriousness of insomnia and its repercussions on the academic and professional life of university students, the purpose of this study was to: 1) Assess SDs in students at the Campus of Medical Sciences (CMS) at Saint-Joseph University in Lebanon, as well as socio-demographic factors associated with insomnia, sleep quality, daytime sleepiness and anxiety; 2) Assess the relationship between different aspects of sleep (insomnia, quality of sleep and sleepiness) and anxiety. Our hypothesis were that SDs could be a major health concern in university students, and that examining anxiety using a simple tool (GAD-7) is primordial when exploring students sleep patterns.

## Materials and Methods

### Ethical considerations

The protocol of the study was approved by the ethics committee of Saint-Joseph University (Ref.USJ-2013-33, July 2013). Informed consent was obtained from all individuals participating in the study.

### Survey procedure and sampling

Our study was a cross-sectional questionnaire-based survey conducted among students of three faculties: medicine, dentistry and pharmacy at Saint-Joseph University, from September 2013 to May 2014 (9 months). Inclusion criteria were: students aged 18 years and above, willing to participate in the study. Exclusion criteria were: age under 18 years and presence of chronic disease. Students were randomly selected within each class using a random number table to ensure the representativeness of the sample. This random selection was proportional to the number of students in each class. Students selected were approached by two trained research assistants usually at the end of their courses before leaving the classroom.

### Data collection

Data were collected during a face-to-face interview using a self-administered standardized survey tool based on four internationally validated and reliable questionnaires, namely the Insomnia Severity Index [17], the Pittsburgh Sleep Quality Index (PSQI) [18], the Epworth Sleepiness Scale [19] and the Generalized Anxiety Disorder 7-item scale (GAD-7) [20]. The duration of interviews ranged from 10 to 20 minutes.

### Questionnaire survey

Personal data about age, gender and faculty were collected. The ISI is a 7-item self-report questionnaire assessing the nature, severity, and impact of insomnia. The evaluated domains are: severity of sleep onset, sleep maintenance, early morning awakening problems, sleep dissatisfaction, interference of sleep difficulties with daytime functioning, perception of sleep difficulties by others, and distress caused by the sleep difficulties. A 5-point Likert scale was used to rate each item (0 to 4 where 0 indicates no problem and 4 corresponds to a very severe problem), yielding a total score ranging from 0 to 28. The total score was interpreted as follows: absence of insomnia (0–7); sub-clinical (mild) insomnia (8–14); moderate insomnia (15–21); and severe insomnia (22–28). Furthermore, clinically significant insomnia was detected when the total score was >14 [21, 22].

The PSQI is a 19-items questionnaire evaluating sleep quality and disturbances over the past month. The first four items are open questions, whereas items 5 to 19 are rated on a 4-point Likert scale. Individual items scores yield seven components (sleep disturbance, overall sleep quality, sleep latency, duration of sleep, daytime dysfunction due to sleepiness, sleep efficiency, and need for medicines to sleep). A total score, ranging from 0 to 21, was obtained by adding the seven component scores. Some studies stated that a score < 5 suggests a good sleep quality [23]. However, the vast majority of studies involving the PSQI state clearly that a score > 5 suggests poor sleep quality whereas a score ≤ 5 suggests a good sleep quality [18, 24, 25]. In our study, we chose the latest PSQI score interpretation.

The ESS is a self-administered questionnaire with eight questions. Each participant rated on a 4-point scale (0–3) his general level of daytime sleepiness, or the average sleep propensity in daily life. The total ESS score was the sum of eight item-scores and ranged between 0 and 24. The higher the score, the higher is the person’s level of daytime sleepiness, with significant sleepiness when the score was > 10 [24, 25]. Finally, GAD-7 is a 7-item instrument that assesses generalized anxiety severity. Each item was scored 0 to 3, providing a 0 to 21 severity score (0–4: normal; 5–9: mild anxiety; 10–14: moderate anxiety and 15–21: severe anxiety), with clinically significant anxiety detected when the total score was 10 or above [20, 26].

### Data analysis

The statistical analysis was carried out using SPSS software for Windows (version 19, Chicago, IL, USA). The significance level was set at 5%. Sample characteristics were summarized using the mean and the standard deviation (SD) for continuous variables and percentage for categorical variables. Insomnia prevalence rate was calculated using descriptive data, along with its corresponding 95% confidence interval (CI). For statistical comparison, analysis of variance or Student’s *t*-tests were used for continuous variables, Chi-square test (χ^2^) or Fisher Exact test were used for categorical variables and Spearman correlation coefficient test for correlations.

## Results

### Socio-demographic characteristics of the participants

A total of 515 students were approached to participate in the study, of whom 462 (89.7%) consented. Our study population comprised 140 (31.3%) male and 322 (69.7%) female students. Age ranged between 18 and 30 years (mean 21.2±1.8 years). The sample included 154 students from the Faculty of medicine (FM), 108 from the Faculty of dentistry (FD) and 200 from the Faculty of pharmacy (FP).

### Insomnia prevalence and severity (ISI)

The presence of insomnia was evaluated according to the ISI questionnaire. Prevalence of clinical insomnia was 10.6% with a 95% confidence interval ranging between 7.8 and 13.4%. The mean ISI score of the sample was 10.06±3.76. Table 1 displays the proportions of participants who endorsed each item response to the ISI questionnaire. Among 462 students, 129 (27.9%) had no insomnia (ISI 0–7), 284 (61.5%) had sub-threshold insomnia (ISI 8–14) and 49 (10.6%) had clinically significant insomnia (ISI > 14). Of these, 48 had moderate and only one had severe insomnia. Neither gender nor faculty was significantly associated with ISI score. The association between participants’ age and ISI score did not reach statistical significance (*p* = 0.064); however, the year of study was found to be statistically significantly associated with the ISI score (*p* = 0.041), clinical insomnia being statistically significantly more frequent in first year students.

**Table 1:** Insomnia Severity Index items: numbers (proportions) of participants endorsing each response (N = 462).

Items of the ISI*	0	1	2	3	4
1. Difficulty falling asleep	147(31.8%)	181(39.2%)	95(20.6%)	31(6.7%)	8(1.7%)
2. Difficulty staying asleep	183(39.6%)	169(36.6%)	83(18.0%)	23(5.0%)	4(0.9%)
3. Early morning awakenings	90(19.5%)	118(25.5%)	130(28.1%)	81(17.5%)	43(9.3%)
4. Sleep dissatisfaction	45(9.7%)	77(16.7%)	237(51.3%)	64(13.9%)	39(8.4%)
5. Interference of sleep problems with daytime functioning	44(9.5%)	114(24.7%)	141(30.5%)	133(28.8%)	30(6.5%)
6. Perception of sleep difficulties by others	106(22.9%)	158(34.2%)	139(30.1%)	53(11.5%)	6(1.3%)
7. Preoccupation and distress caused by sleep difficulties	151(32.7%)	162(35.1%)	107(23.2%)	34(7.4%)	8(1.7%)

*For items 1 to 3, 0 = no problem, 1 = mild, 2 = moderate, 3 = severe and 4 = very severe. **For item 4**, 0 = very satisfied, 1 = satisfied, 2 = neutral, 3 = dissatisfied and 4 = very dissatisfied. **For items 5 to 7**, 0 = not at all, 1 = a little, 2 = somewhat, 3 = much and 4 = very much.

### Subjective Sleep Quality (PSQI)

The mean PSQI score was 5.14±2.84 and 287 (62.9%) participants were good sleepers. Age, gender, faculty and year of study did not differ significantly between good and poor sleepers (*p*>0.05). However, sleep duration and sleep disturbance subscales differed significantly between the three faculties (Table 2). Sleep duration was shortest for students of the FM while sleep disturbance was more pronounced for the FD students. Subjective sleep quality, sleep duration, sleep disturbance and daytime dysfunction were significantly associated with age and year of study. Only sleep efficiency subscale was significantly different between male and female participants (*p* = 0.039), worse for females.

**Table 2:** Socio-demographic factors associated with the different domains of the PSQI questionnaire (N = 456*).

PSQI subscales							
	Subjective Sleep Quality	Sleep Latency	Duration	Sleep Efficiency	Sleep Disturbance	Medication	Daytime Dysfunction
**Faculty**							
FM	1.03±0.75	0.84±0.91	0.83±0.94	0.28±0.63	0.94±0.48	0.10±0.41	1.16±0.87
(n = 153)							
FD	1.12±0.85	0.82±0.96	0.66±0.79	0.23±0.64	1.09±0.59	0.21±0.64	1.21±0.96
(n = 107)							
FP	1.12±0.85	0.82±0.96	0.66±0.79	0.23±0.64	1.09±0.59	0.21±0.64	1.21±0.96
(n = 196)							
*p*	.585	.951	**.014**	.319	**.016**	.187	.274
**Gender**							
Male	1.01±0.75	0.95±0.93	0.70±0.86	0.20±0.53	0.99±0.50	0.17±0.56	1.06±0.83
(n = 139)							
Female	1.10±0.77	0.80±0.89	0.66±0.87	0.34±0.73	1.05±0.48	0.12±0.45	1.16±0.86
(n = 317)							
*p*	.211	.100	.698	**.039**	.246	.326	.218
**Year of study**							
First	1.28±0.81	0.93±1.01	0.72±0.86	0.28±0.71	1.16±0.59	0.10±0.42	1.33±0.84
(n = 122)							
Second	1.00±0.71	0.93±0.86	0.60±0.82	0.42±0.71	1.04±0.42	0.33±0.83	1.07±0.88
(n = 57)							
Third	1.21±0.77	0.79±0.90	0.82±0.91	0.23±0.63	1.06±0.48	0.14±0.44	1.18±0.81
(n = 103)							
Fourth	1.10±0.69	0.87±0.78	0.71±1.03	0.32±0.67	0.98±0.46	0.10±0.39	0.94±0.88
(n = 62)							
Fifth	0.76±0.66	0.70±0.82	0.46±0.69	0.33±0.72	0.93±0.36	0.07±0.29	1.09±0.82
(n = 89)							
Sixth	0.62±0.65	0.69±0.75	0.38±0.65	0.08±0.28	0.77±0.44	0.00±0.00	0.62±0.87
(n = 13)							
Seventh	0.80±0.63	1.30±1.34	1.10±1.10	0.50±0.71	0.80±0.45	0.30±0.67	0.80±0.63
(n = 10)							
*p*	**<0.0001**	0.301	**0.040**	0.495	**0.005**	**0.025**	**0.008**
**Correlation**	-0.224	-0.068	-0.110	0.024	-0.156	-0.010	-0.158
**Coefficient**							
*p*	**<0.0001**	0.155	**0.022**	0.620	**0.001**	0.842	**0.001**

*Presence of missing values; the numbers in bold represent statistically significant results.

The proportion of poor sleepers was higher in participants with clinical insomnia (*p* = 0.001); however, PSQI and ESS were not significantly associated (*p*>0.05).

### Anxiety (GAD-7)

Anxiety was evaluated using the GAD-7 tool. The mean GAD-7 score was 7.01±4.81. Table 3 displays the proportion of participants who endorsed each item response to the GAD-7 questionnaire. Our study showed that 37.1% of the participants do not suffer from anxiety, 33.7% have mild anxiety, 21.6% moderate and 7.1% severe anxiety. Overall, 28.7% of students presented clinically significant anxiety. Anxiety was not significantly associated with any of the socio-demographic characteristics of the participants (*p*>0.05).

**Table 3:** GAD-7 items with corresponding numbers (proportions) of participants endorsing each score (N = 436*).

Over the last 2 weeks, how often have you been bothered by the following problem?				
	Not at all	Several days	More than half the days	Nearly everyday
1. Feeling nervous, anxious or on edge	68(15.6%)	210(48.2%)	98(22.5%)	60(13.8%)
2. Not being able to stop or control worrying	114(26.1%)	192(44.0%)	89(20.4%)	41(9.4%)
3. Worrying too much about different things	185(42.4%)	141(32.3%)	70(16.1%)	40(9.2%)
4. Trouble relaxing	165(37.8%)	172(39.4%)	72(16.5%)	27(6.2%)
5. Being so restless that it is hard to sit still	190(43.6%)	172(39.4%)	57(13.1%)	17(3.9%)
6. Becoming easily annoyed or irritable	116(26.6%)	203(46.6%)	86(19.7%)	31(7.1%)
7. Feeling afraid as if something awful might happen	183(42.0%)	158(36.2%)	66(15.1%)	29(6.7%)

*Presence of missing values.

### Daytime sleepiness (ESS)

Daytime sleepiness of the participants was evaluated using the ESS tool. The mean ESS score was 7.71±4.18. Of all participants, 23.9% scored above 10 on the ESS and were considered to have excessive daytime sleepiness. One item was associated with a high chance of dozing in nearly 30% of the students: *“lying down to rest in the afternoon when circumstances permit”* (Table 4). Daytime sleepiness was not significantly associated with any of the socio-demographic characteristics of the participants (*p*>0.05).

**Table 4:** Epworth Sleepiness Scale: items with corresponding numbers (proportions) of participants endorsing each score (N = 436*).

ESS	No chance of dozing	Slight chance of dozing	Moderate chance of dozing	High chance of dozing
Sitting and reading	103(23.6%)	134(30.7%)	162(37.2%)	37(8.5%)
Watching TV	123(28.2%)	158(36.2%)	121(27.8%)	34(7.8%)
Sitting inactive in a public place (e.g a theater or a meeting)	176(40.4%)	156(35.8%)	83(19.0%)	21(4.8%)
As a passenger in a car for an hour without break	151(34.6%)	136(31.2%)	115(26.4%)	34(7.8%)
Lying down to rest in the afternoon when circumstances permit	45(10.3%)	94(21.6%)	169(38.8%)	128(29.4%)
Sitting and talking to someone	323(74.1%)	91(20.9%)	19(4.4%)	3(.7%)
Sitting quietly after a lunch without alcohol	238(54.6%)	115(26.4%)	63(14.4%)	20(4.6%)
In a car, while stopped for a few minutes in traffic	300(68.8%)	97(22.2%)	31(7.1%)	8(1.8%)

*Presence of missing values.

Excessive daytime sleepiness was significantly more frequent among participants with clinical insomnia (*p* = 0.031) than those with no clinical insomnia.

### Associations between anxiety (GAD-7) and ISI, PSQI or ESS

Clinically significant anxiety was significantly more frequent in students suffering from clinical insomnia (*p* = 0.006) and in poor sleepers (*p* = 0.003). Moreover, 50.8% of the participants with clinically significant anxiety presented excessive daytime sleepiness, versus 30.9% of those with no clinically significant anxiety (*p*<0.0001) (Table 5).

**Table 5:** Associations between anxiety (GAD-7) on one hand and ISI, PSQI or ESS on the other hand (number and percentage of participants are shown).

	No clinically significant anxiety	Clinically significant anxiety	-p-value
**ISI**			**0.006**
No clinically significant insomnia	99(31.8%)	24(19.2%)	
Sub-threshold insomnia	186(59.8%)	81(64.8%)	
Clinical insomnia (moderate severity)	25(8.0%)	20(16.0%)	
Clinical insomnia (severe)	1(.3%)	0(.0%)	
*Total*	*311(100*.*0%)*	*125(100*.*0%)*	
**PSQI**			**0.003**
Good sleeper	243(79.9%)	81(66.4%)	
Poor sleeper	61(20.1%)	41(33.6%)	
*Total*	*304(100*.*0%)*	*122(100*.*0%)*	
**ESS**			**<0.0001**
Normal	215(69.1%)	61(49.2%)	
Excessive daytime sleepiness	96(30.9%)	63(50.8%)	
*Total*	*311(100*.*0%)*	*124(100*.*0%)*	

The numbers in bold represent statistically significant results.

## Discussion

Insomnia is one of the most common SDs, with prevalence between 10% and 40% across studies [27, 28]. Prevalence of sleep disorders among young adults is increasing globally and in adolescents aged 13–16, prevalence of insomnia was reported to be 11% [29].

Our study revealed that 10.6% of participants suffered from clinically significant insomnia. These results are consistent with the nature of the sample studied (young students) and is comparable with what is reported in young adults aged 20 to 29 (9.1%) [30] and in college students (12–13%) [31].

Quality of sleep is another important parameter to explore, since it was associated with quality of life in many studies [32, 33]. Students classified as poor-sleepers experienced more problems with physical and psychological health. Improving sleep quality has been shown to decrease the incidence of chronic diseases such as major depressive disorders, psychosis, addictions, cardiovascular, metabolic and inflammatory disease risks [34]. Our study revealed that a considerable number (37.1%) of participants are poor sleepers and poor sleep quality was significantly related to the presence of clinical insomnia (*p* = 0.001).

Excessive daytime sleepiness is a symptom with high clinical and public health importance because of its association with increased risk for accidents, decreased productivity, metabolic syndrome, diabetes and impaired quality of life [35, 36]. Insomnia symptoms and anxiety were associated with the subsequent occurrence of excessive daytime sleepiness in one study [35]. However, excessive daytime sleepiness was associated with depression, but not anxiety in another study [37]. Our results showed a significant association between anxiety and excessive daytime sleepiness: 50.8% of the participants with clinically significant anxiety presented excessive daytime sleepiness, versus 30.9% of the participants with no clinically significant anxiety (*p*<0.0001). Furthermore, excessive daytime sleepiness was significantly more frequent in students with clinical insomnia (*p* = 0.031).This latest finding is of primary importance since previous publications assessing the relationship between insomnia and excessive daytime sleepiness show conflicting findings [35] [38].

Furthermore, our results showed no association between ESS and PSQI scores, which is a finding reported previously [18, 25]. The explanation could be that these two questionnaires evaluate different aspects of sleep [24].

Overall, 28.7% of students presented clinically significant anxiety and our results are comparable to what is previously reported among university students [39].

One of the most robust associations between sleep disruption and emotional functioning involves the role of anxiety [40, 41], and insomnia increases the likelihood of developing a mood or anxiety disorder [42]. In terms of public health, insomnia co-morbid with anxiety or depression gives rise to more complications and greater burdens than does each disorder alone [43] and treating anxiety improves co-morbid insomnia symptoms [42].

Our results showed that anxiety was significantly associated with insomnia as well as with sleep quality and daytime sleepiness.

### Strength and limitations

Our findings should be interpreted in the context of the study’s design and limitations. The results of our survey rely on self-reported behavior. Self-reporting questionnaires remain the most widely used tools in community surveys [44, 45]. The self-report method reflects the interviewee’s own perspective, which may be more suitable for reporting subjective disorders. The questionnaires were formulated in a “multiple-choice” and scale pattern to facilitate response and have shorter interview duration in order to avoid disturbing the students, in the hope that the simplicity of the questionnaire would make it easier for the respondents to give accurate informations. PSQI subscales show a significant difference between faculties and this probable bias needs to be highlighted. Sleep hygiene and sleep schedules have not been examined and need to be explored in future studies since they are important determinant of behavioral insomnia in college and university students [31]. Furthermore, anxiety and not depression was assessed in our study. However, since the prevalence of anxiety in university students is much higher than depression [39] and since no causal relationship between anxiety and insomnia is proposed, we are confident that this limitation did not influence our results. Finally, comorbidities and use of drugs were not examined since the presence of any chronic disease was among the exclusion criteria of this study.

Notwithstanding these limitations, the findings observed in this study are important and warrant further investigations.

To the best of our knowledge, this was the first study that examines three different aspects of sleep health in university students: insomnia, quality of sleep, and excessive daytime sleepiness. It is also the first that assesses the relationships between these three SDs and anxiety. ISI and PSQI were combined in numerous studies and some others combine them with ESS or to GAD-7 but none with both.

In conclusion, our study explored several aspects of sleep parameters in university students together with anxiety assessment. The magnitude of sleep perturbation, in this sample of university students demonstrates the importance of examining sleep health in this population. Anxiety is not only associated with insomnia but also with poor sleep quality and with excessive daytime sleepiness among these students. These findings underline the importance of anxiety evaluation when assessing sleep in similar populations. Due to the bidirectional pathways between anxiety and sleep, interventions aiming at breaking the circle of sleep-affecting anxiety and anxiety affecting sleep are needed. Anxiety management, along with other measures such as sleep-hygiene and stress management measures could improve students’ sleep.

## References

[1] National Center On Sleep Disorders Research. National Institutes Of Health Sleep Disorders Research Plan. National Institutes of Health (11 2011). Available: https://www.nhlbi.nih.gov/health/prof/sleep/201101011NationalSleepDisordersResearchPlanDHHSPublication11-7820.pdf, Accessed 23 January 2015.

[2] Chung KF, Yeung WF, Ho FY, Yung KP, Yu YM, Kwok CW. Cross-cultural and comparative epidemiology of insomnia: the Diagnostic and statistical manual (DSM), International classification of diseases (ICD) and International classification of sleep disorders (ICSD). Sleep Med. 2015;16(4):477–82. 10.1016/j.sleep.2014.10.018 25761665

[3] Roth T. Insomnia: definition, prevalence, etiology, and consequences. J Clin Sleep Med. 2007;3(5 Suppl):S7–10. 17824495 PMC1978319

[4] Ohayon MM, Riemann D, Morin C, Reynolds CF 3rd. Hierarchy of insomnia criteria based on daytime consequences. Sleep Med. 2012;13(1):52–7. 10.1016/j.sleep.2011.06.010 22036602 PMC3249005

[5] American psychiatric association, diagnostic and statistical manual of mental disorders DSM-5 5th ed: American Psychiatric publishing; 2013.

[6] Banks S, Dinges DF. Behavioral and physiological consequences of sleep restriction. J Clin Sleep Med. 2007;3(5):519–28. 17803017 PMC1978335

[7] Ohayon MM. Epidemiology of insomnia: what we know and what we still need to learn. Sleep Med Rev. 2002;6(2):97–111. 12531146 10.1053/smrv.2002.0186

[8] Goldman-Mellor S, Gregory AM, Caspi A, Harrington H, Parsons M, Poulton R, et al Mental health antecedents of early midlife insomnia: evidence from a four-decade longitudinal study. Sleep. 2014;37(11):1767–75. 10.5665/sleep.4168 25364072 PMC4196060

[9] Forbes EE, Bertocci MA, Gregory AM, Ryan ND, Axelson DA, Birmaher B, et al Objective sleep in pediatric anxiety disorders and major depressive disorder. J Am Acad Child Adolesc Psychiatry. 2008;47(2):148–55. 10.1097/chi.0b013e31815cd9bc 18176336 PMC2674333

[10] Storch EA, Murphy TK, Lack CW, Geffken GR, Jacob ML, Goodman WK. Sleep-related problems in pediatric obsessive-compulsive disorder. J Anxiety Disord. 2008;22(5):877–85. 17951025 10.1016/j.janxdis.2007.09.003 PMC2413417

[11] Royal college of psychiatrists, Mental health of students in higher education. London: 2011.

[12] Jansson M, Linton SJ. The development of insomnia within the first year: a focus on worry. British journal of health psychology. 2006;11(Pt 3):501–11. 16870058 10.1348/135910705X57412

[13] Morphy H, Dunn KM, Lewis M, Boardman HF, Croft PR. Epidemiology of insomnia: a longitudinal study in a UK population. Sleep. 2007;30(3):274–80. 17425223

[14] Jansson-Frojmark M, Lindblom K. A bidirectional relationship between anxiety and depression, and insomnia? A prospective study in the general population. J Psychosom Res. 2008;64(4):443–9. 10.1016/j.jpsychores.2007.10.016 18374745

[15] Johnson EO, Roth T, Breslau N. The association of insomnia with anxiety disorders and depression: exploration of the direction of risk. J Psychiatr Res. 2006;40(8):700–8. 16978649 10.1016/j.jpsychires.2006.07.008

[16] Monti JM, Monti D. Sleep in schizophrenia patients and the effects of antipsychotic drugs. Sleep Med Rev. 2004;8(2):133–48. 15033152 10.1016/S1087-0792(02)00158-2

[17] Morin CM, Belleville G, Belanger L, Ivers H. The Insomnia Severity Index: psychometric indicators to detect insomnia cases and evaluate treatment response. Sleep. 2011;34(5):601–8. 21532953 10.1093/sleep/34.5.601 PMC3079939

[18] Buysse DJ, Reynolds CF 3rd, Monk TH, Berman SR, Kupfer DJ. The Pittsburgh Sleep Quality Index: a new instrument for psychiatric practice and research. Psychiatry Res. 1989;28(2):193–213. 2748771 10.1016/0165-1781(89)90047-4

[19] Johns MW. A new method for measuring daytime sleepiness: the Epworth sleepiness scale. Sleep. 1991;14(6):540–5. 1798888 10.1093/sleep/14.6.540

[20] Spitzer RL, Kroenke K, Williams JB, Lowe B. A brief measure for assessing generalized anxiety disorder: the GAD-7. Arch Intern Med. 2006;166(10):1092–7. 16717171 10.1001/archinte.166.10.1092

[21] Cho YW, Song ML, Morin CM. Validation of a Korean version of the insomnia severity index. J Clin Neurol. 2014;10(3):210–5. 10.3988/jcn.2014.10.3.210 25045373 PMC4101097

[22] Gagnon C, Belanger L, Ivers H, Morin CM. Validation of the Insomnia Severity Index in primary care. J Am Board Fam Med. 2013;26(6):701–10. 10.3122/jabfm.2013.06.130064 24204066

[23] Murthy VS, Nayak AS. Assessment of sleep quality in post-graduate residents in a tertiary hospital and teaching institute. Ind Psychiatry J. 2014;23(1):23–6. 10.4103/0972-6748.144952 25535441 PMC4261209

[24] Buysse DJ, Hall ML, Strollo PJ, Kamarck TW, Owens J, Lee L, et al Relationships between the Pittsburgh Sleep Quality Index (PSQI), Epworth Sleepiness Scale (ESS), and clinical/polysomnographic measures in a community sample. J Clin Sleep Med. 2008;4(6):563–71. 19110886 PMC2603534

[25] Mondal P, Gjevre JA, Taylor-Gjevre RM, Lim HJ. Relationship between the Pittsburgh Sleep Quality Index and the Epworth Sleepiness Scale in a sleep laboratory referral population. Nat Sci Sleep. 2013;5:15–21. 10.2147/NSS.S40608 23620689 PMC3630984

[26] Kroenke K, Spitzer RL, Williams JB, Monahan PO, Lowe B. Anxiety disorders in primary care: prevalence, impairment, comorbidity, and detection. Ann Intern Med. 2007;146(5):317–25. 17339617 10.7326/0003-4819-146-5-200703060-00004

[27] Blais FC, Morin CM, Boisclair A, Grenier V, Guay B. [Insomnia. Prevalence and treatment of patients in general practice]. Can Fam Physician. 2001;47:759–67. 11340757 PMC2018415

[28] Leger D, Partinen M, Hirshkowitz M, Chokroverty S, Hedner J. Characteristics of insomnia in a primary care setting: EQUINOX survey of 5293 insomniacs from 10 countries. Sleep Med. 2010;11(10):987–98. 10.1016/j.sleep.2010.04.019 21093363

[29] Johnson EO, Roth T, Schultz L, Breslau N. Epidemiology of DSM-IV insomnia in adolescence: lifetime prevalence, chronicity, and an emergent gender difference. Pediatrics. 2006;117(2):e247–56. 16452333 10.1542/peds.2004-2629

[30] Thomas SJ, Lichstein KL, Taylor DJ, Riedel BW, Bush AJ. Epidemiology of bedtime, arising time, and time in bed: analysis of age, gender, and ethnicity. Behav Sleep Med. 2014;12(3):169–82. 10.1080/15402002.2013.778202 23574553

[31] Gellis LA, Park A, Stotsky MT, Taylor DJ. Associations between sleep hygiene and insomnia severity in college students: cross-sectional and prospective analyses. Behav Ther. 2014;45(6):806–16. 10.1016/j.beth.2014.05.002 25311289

[32] Leistner SM, Klotsche J, Dimopoulou C, Athanasoulia AP, Roemmler-Zehrer J, Pieper L, et al Reduced sleep quality and depression associate with decreased quality of life in patients with pituitary adenomas. Eur J Endocrinol. 2015. 10.1530/EJE-14-0941 25792374

[33] Sekercioglu N, Curtis B, Murphy S, Barrett B. Sleep quality and its correlates in patients with chronic kidney disease: a cross-sectional design. Ren Fail. 2015:1–6. 10.3109/0886022X.2015.1024555 25782921

[34] Carroll JE, Seeman TE, Olmstead R, Melendez G, Sadakane R, Bootzin R, et al Improved sleep quality in older adults with insomnia reduces biomarkers of disease risk: Pilot results from a randomized controlled comparative efficacy trial. Psychoneuroendocrinology. 2015;55:184–92. 10.1016/j.psyneuen.2015.02.010 25770704 PMC4422640

[35] Hasler G, Buysse DJ, Gamma A, Ajdacic V, Eich D, Rossler W, et al Excessive daytime sleepiness in young adults: a 20-year prospective community study. J Clin Psychiatry. 2005;66(4):521–9. 15816796 10.4088/jcp.v66n0416

[36] Hayley AC, Williams LJ, Kennedy GA, Berk M, Brennan SL, Pasco JA. Excessive daytime sleepiness and metabolic syndrome: a cross-sectional study. Metabolism. 2015;64(2):244–52. 10.1016/j.metabol.2014.09.011 25441252

[37] Hayley AC, Williams LJ, Berk M, Kennedy GA, Jacka FN, Pasco JA. The relationship between excessive daytime sleepiness and depressive and anxiety disorders in women. Aust N Z J Psychiatry. 2013;47(8):772–8. 10.1177/0004867413490036 23677847

[38] Taylor DJ, Bramoweth AD, Grieser EA, Tatum JI, Roane BM. Epidemiology of insomnia in college students: relationship with mental health, quality of life, and substance use difficulties. Behav Ther. 2013;44(3):339–48. 10.1016/j.beth.2012.12.001 23768662

[39] Shamsuddin K, Fadzil F, Ismail WS, Shah SA, Omar K, Muhammad NA, et al Correlates of depression, anxiety and stress among Malaysian university students. Asian journal of psychiatry. 2013;6(4):318–23. 10.1016/j.ajp.2013.01.014 23810140

[40] Alfano CA, Pina AA, Zerr AA, Villalta IK. Pre-sleep arousal and sleep problems of anxiety-disordered youth. Child Psychiatry Hum Dev. 2010;41(2):156–67. 10.1007/s10578-009-0158-5 19680805 PMC2818382

[41] Gregory AM, O'Connor TG. Sleep problems in childhood: a longitudinal study of developmental change and association with behavioral problems. J Am Acad Child Adolesc Psychiatry. 2002;41(8):964–71. 12162632 10.1097/00004583-200208000-00015

[42] Mason EC, Harvey AG. Insomnia before and after treatment for anxiety and depression. J Affect Disord. 2014;168:415–21. 10.1016/j.jad.2014.07.020 25108278

[43] Belleville G, Cousineau H, Levrier K, St-Pierre-Delorme ME. Meta-analytic review of the impact of cognitive-behavior therapy for insomnia on concomitant anxiety. Clin Psychol Rev. 2011;31(4):638–52. 10.1016/j.cpr.2011.02.004 21482322

[44] Armstrong D, Dregan A. A population-based investigation into the self-reported reasons for sleep problems. PLoS One. 2014;9(7):e101368 10.1371/journal.pone.0101368 24983754 PMC4077805

[45] Habte-Gabr E, Wallace RB, Colsher PL, Hulbert JR, White LR, Smith IM. Sleep patterns in rural elders: demographic, health, and psychobehavioral correlates. J Clin Epidemiol. 1991;44(1):5–13. 1986057 10.1016/0895-4356(91)90195-f

